# Respiratory mechanics and cerebral blood flow during heat‐induced hyperventilation and its voluntary suppression in passively heated humans

**DOI:** 10.14814/phy2.13967

**Published:** 2019-01-13

**Authors:** Bun Tsuji, Yuta Hoshi, Yasushi Honda, Naoto Fujii, Yosuke Sasaki, Stephen S. Cheung, Narihiko Kondo, Takeshi Nishiyasu

**Affiliations:** ^1^ Department of Health Sciences Prefectural University of Hiroshima Hiroshima Japan; ^2^ Faculty of Health and Sport Sciences University of Tsukuba Tsukuba City Ibaraki Japan; ^3^ Department of Kinesiology Brock University St. Catharines Canada; ^4^ Graduate School of Human Development and Environment Kobe University Kobe Japan

**Keywords:** Cerebral blood flow, hyperpnea, hyperthermia, voluntary control of breathing

## Abstract

We investigated whether heat‐induced hyperventilation can be voluntarily prevented, and, if so, how this modulates respiratory mechanics and cerebral blood flow in resting heated humans. In two separate trials, 10 healthy men were passively heated using lower body hot‐water immersion and a water‐perfused garment covering their upper body (both 41°C) until esophageal temperature (*T*_es_) reached 39°C or volitional termination. In each trial, participants breathed normally (normal‐breathing) or voluntarily controlled minute ventilation (*V*_E_) at a level equivalent to that observed after 5 min of heating (controlled‐breathing). Respiratory gases, middle cerebral artery blood velocity (MCAV), work of breathing, and end‐expiratory and inspiratory lung volumes were measured. During normal‐breathing, *V*_E_ increased as *T*_es_ rose above 38.0 ± 0.3°C, whereas controlled‐breathing diminished the increase in *V*_E_ (*V*_E_ at *T*_es_ = 38.6°C: 25.6 ± 5.9 and 11.9 ± 1.3 L min^−1^ during normal‐ and controlled‐breathing, respectively, *P *<* *0.001). During normal‐breathing, end‐tidal CO_2_ pressure and MCAV decreased with rising *T*_es_, but controlled‐breathing diminished these reductions (at *T*_es_ = 38.6°C, 24.7 ± 5.0 vs. 39.5 ± 2.8 mmHg; 44.9 ± 5.9 vs. 60.2 ± 6.3 cm sec^−1^, both *P *<* *0.001). The work of breathing correlated positively with changes in *V*_E_ (*P *<* *0.001) and was lower during controlled‐ than normal‐breathing (16.1 ± 12.6 and 59.4 ± 49.5 J min^−1^, respectively, at heating termination, *P *= 0.013). End‐expiratory and inspiratory lung volumes did not differ between trials (*P *= 0.25 and 0.71, respectively). These results suggest that during passive heating at rest, heat‐induced hyperventilation increases the work of breathing without affecting end‐expiratory lung volume, and that voluntary control of breathing can nearly abolish this hyperventilation, thereby diminishing hypocapnia, cerebral hypoperfusion, and increased work of breathing.

## Introduction

Hyperthermia is known to cause cerebral hypoperfusion at rest (Fan et al. 2008; Fujii et al. 2008a; Low et al. 2008; Brothers et al. 2009; Nelson et al. 2011) and during exercise (Nybo and Nielsen 2001; Hayashi et al. 2011). This effect may cause elevations in brain temperature (Nybo et al. 2002) and central fatigue (Ross et al. 2012), which could contribute to the development of heat‐related illness and to decreased exercise performance in the heat. Thus, reversal of the reduced cerebral blood flow during hyperthermia could reduce the risk of heat‐related illness and improve exercise performance in the heat.

Hyperthermia also leads to increases in minute ventilation (*V*_E_) at rest (Saxton 1975; Cabanac and White 1995; Fujii et al. 2015) and during exercise (White and Cabanac 1996; Nybo and Nielsen 2001; Hayashi et al. 2011; Tsuji et al. 2012b). This hyperventilation results in excessive elimination of CO_2_ from the body, leading to reductions in arterial CO_2_ pressure (PaCO_2_) (hypocapnia). Moreover, the hyperventilation‐induced hypocapnia contributes to cerebral hypoperfusion during hyperthermia at rest (Fujii et al. 2008a; Brothers et al. 2009; Nelson et al. 2011) and during exercise (Rasmussen et al. 2006; Hayashi et al. 2011). Thus, if one could suppress heat‐induced hyperventilation, they may be able to substantially reverse hyperthermia‐induced cerebral hypoperfusion.

We recently reported that, during prolonged exercise in the heat, heat‐induced hyperventilation can be voluntarily suppressed using audiovisual feedback so that PaCO_2_ is maintained at the eucapnic level throughout the exercise (Tsuji et al. 2015). This largely reverses the heat‐induced decreases in middle cerebral artery blood flow velocity (MCAV; an index of anterior cerebral blood flow) otherwise seen (Tsuji et al. 2015). However, whether heat‐induced hyperventilation occurring during passive heating at rest can also be suppressed through voluntary control of breathing remains unknown. The heat‐induced hyperventilation and the resultant hypocapnia at rest are three times greater than during exercise (Fujii et al. 2008b; Tsuji et al. 2012a). Because respiratory effort and dyspnea increase as the magnitude of ventilation suppression increases (Chonan et al. 1990), voluntarily suppressing hyperventilation and hypocapnia throughout passive heating at rest could be more challenging than during exercise.

Heat‐induced hyperventilation is initiated during passive heating when core temperature exceeds ~38.0–38.5°C. Thereafter, *V*_E_ increases at a rate of ~20–30 L min^−1^ of *V*_E_ per 1°C rise in core temperature (Cabanac and White 1995; Fujii et al. 2008b; Tsuji et al. 2012a). Although these features of hyperventilation have been extensively reported, available information on respiratory mechanics, such as lung volume and work of breathing during hyperthermia at rest remains unknown. During maximal incremental exercise accompanied by increases in core temperature, end‐expiratory lung volume decreases below the resting level while tidal volume (*V*_T_) increases (Henke et al. 1988; Guenette et al. 2007). Heat‐induced hyperventilation at rest is due to increases in both *V*_T_ and respiratory frequency (*f*) (Gaudio and Abramson 1968; Baker et al. 1996; Fujii et al. 2008b; Tsuji et al. 2012a), though it remains uncertain whether the lung inflates or deflates more or less than normothermic resting levels. If heat‐induced hyperventilation at rest is accompanied by changes in end‐expiratory lung volume, work of breathing would be greater than expected, since work of breathing increases as the end‐expiratory lung volume increases or decreases from the normal resting level (Butler and Arnott 1955).

In this study, therefore, we examined whether one can voluntarily suppress heat‐induced hyperventilation during passive heating at rest, and how this suppression modulates PaCO_2_ and MCAV. We hypothesized that voluntarily suppressing the heat‐induced hyperventilation in healthy men, if possible, would largely alleviate hypocapnia and cerebral hypoperfusion, both of which occur during passive heating. We also evaluated how heat‐induced hyperventilation and voluntary suppression of this response modulate respiratory mechanics, such as lung volume and the work of breathing.

## Methods

### Participants

Ten healthy males [age: 24 ± 2 years, height: 173 ± 4 cm, weight: 66 ± 8 kg] volunteered to participate in this study. The participants were nonsmokers and were taking no prescription medications. Written informed consent was obtained from each individual before his participation. The present protocol was approved by the Human Subjects Committee of the University of Tsukuba and conformed to the provisions of the Declaration of Helsinki.

### Experimental design

Participants performed two trials wherein they were passively heated with or without controlling breathing. The experimental trials were conducted in a random order and were separated by at least 5 days. The participants were asked to abstain from strenuous exercise, alcohol, and caffeine for 24 h before the experimental testing. To standardize their hydration status, participants drank 500 mL water the night before the experiment and consumed a light meal and 500 mL water 2 h prior to the experiment.

### Procedure

Participants arrived at the laboratory at 0830 h, and body weight was recorded after the voiding of urine. The participants then entered an environmental chamber (ambient temperature = 25°C, relative humidity = 50%) and put on a water‐perfused garment that covered their upper body, except for the head, left forearm, and hands; the hands were sheathed in cotton gloves. The participants sat in a chair situated in a bath (water temperature = 35°C) filled with water up to their iliac crest. The water in the bath was also circulated through the water‐perfused garment. After applying instrumentation, baseline measurements prior to heating (pre) were collected for 10 min while the participants remained sitting. The temperature of the water in the bath, and thus the perfused garment, was then increased to 41°C and maintained at that temperature for the remainder of the experiment. The water temperature was controlled using a heater and was monitored using a thermocouple placed in the bath.

During heating, esophageal temperature (*T*_es_) gradually increased from normothermia, and participants either breathed normally (normal‐breathing trial) or managed to control *f* and *V*_T_, and therefore *V*_E_, at the individual's target level throughout the heating (controlled‐breathing trial). The *V*_T_ and *f* values recorded 5 min into the heating, at which point stable low *f* and *V*_T_ values could be obtained, were used as target values for the controlled‐breathing trial. The target *f* was signaled using a metronome, while the target *V*_T_ was indicated through visual *V*_T_ feedback displayed on a monitor. After 10 min of heating, subjects began voluntarily controlling their breathing and continued until the end of heating. The heating was terminated when *T*_es_ reached 39.0°C or at volitional intolerance, after which the participants were deinstrumented and postheating body weight was recorded.

The participants performed forced vital capacity measurements three times before the heating procedure. In addition, inspiratory capacity maneuvers were performed before and every 5 min during the heating; these maneuvers were used to estimate changes in lung volume.

### Measurements

#### Body temperature

*T*_es_ and skin temperature were measured using copper‐constantan thermocouples and recorded on a computer (Satellite K22, Toshiba, Japan) at 1‐sec intervals via a data logger system (WE7000, Yokogawa, Japan). The thermocouple for measuring *T*_es_ was inserted through the nasal passage to a distance equivalent to one‐fourth of the participant's height. The location of the probe in the esophagus was estimated to be posterior to the lower border of the left atrium (Wenger and Roberts 1980). Mean skin temperature was calculated from the skin temperatures at seven locations weighted as follows: forehead, 7%; forearm, 14%; hand, 5%; foot, 7%; lower leg, 13%; thigh, 19%; and chest, 35% (Hardy and Dubois 1938).

#### Heart rate and blood pressure

Heart rate was recorded every 5 sec using a heart rate monitor (Vantage NV, Polar, Finland). Blood pressure was measured from the upper right arm every 1 min using an automated sphygmomanometer (STBP‐780, Nippon Colin, Japan). Mean arterial pressure (MAP) was calculated as the diastolic pressure plus one‐third of the pulse pressure.

#### Respiratory variables

Inspired and expired gases were analyzed using a metabolic cart (AE310s, Minato Medical Science, Japan). The flow sensor was calibrated using an appurtenant calibration syringe able to deliver a fixed volume (2 L) of air. The O_2_ and CO_2_ sensors were calibrated with room air and reference gases of known concentration (O_2_ 15%, CO_2_ 5%, N_2_ balance). *V*_E_ and *V*_T_ adjusted to BTPS, *f*, oxygen uptake (*V*O_2_), carbon dioxide output (*V*CO_2_), end‐tidal CO_2_ pressure (*P*_ETCO2_), and respiratory exchange ratio were recorded breath‐by‐breath. To calculate the ventilatory mechanics (see Data Analysis for details), the flow signal was recorded via an A/D converter system (Power Lab 16/30, AD Instruments, Japan) at a sampling rate of 200 Hz.

#### Esophageal and intraoral pressures

To estimate the work of breathing, esophageal and intraoral pressures were measured using pressure transducers (Codman Microsensor, Codman), and were recorded on a computer (Satellite J72, Toshiba, Japan) at a sampling rate of 200 Hz via a digital manometer (TCB‐500, Millar instruments) and A/D converter system (Power Lab 16/30, ADInstruments, Japan). The pressure transducer for esophageal pressure was inserted from the nasal passage to a distance equivalent to one‐fourth of the height minus 9 cm, then was further inserted to the stomach, where pressure was changing from negative to positive. Thereafter, the probe was partially withdrawn to a point where the pressure read −1 to −10 cm H_2_O. Another transducer for intraoral pressure was inserted into the oral cavity ~3 cm away from the labium so as not to touch the tongue or hard palate. The pressure transducers were calibrated by immersing them in ~38°C water to depths of 0, 10, 20, 30, and 40 cm.

#### Cerebral blood flow velocity

Middle cerebral artery blood velocity was measured using a transcranial Doppler ultrasound device (WAKI 1‐TC, Atys Medical, France). A 2‐MHz Doppler probe was secured with a customized headband to the left temporal region, and the signal was collected from a depth of 45–60 mm. Prior to the two experiments, the position and angle of the probe were predetermined and imaged using a digital camera. This enabled us to establish similar MCAV values at baseline in the two trials. Cerebral vascular conductance (CVC) was calculated as MCAV divided by mean arterial pressure.

#### Other variables

The participants were instructed to rate the intensity of the sensation of difficulty in breathing (dyspnea) (Chonan et al. 1990) every 5 min using a 10‐point scale (e.g., 1: very easy, 3: moderate, 5: hard, 7: very hard) in nine participants. Body weight was measured using a platform scale with an accuracy of ±10 g (Yamato scale, Japan) before and after the experiment.

### Data analysis

To examine respiratory mechanics during passive heating, five breaths before inspiratory capacity maneuvers were analyzed using flow and pressure signals. One breath, consisting of one inspiration and one expiration, was defined as from initiation of inspiration to the end of expiration. The work of breathing was calculated as the area under the pressure–volume loop multiplied by *f*, with pressure calculated as the difference between the intraoral and esophageal pressures (Otis et al. 1950). End‐expiratory lung volume was calculated by subtracting the inspiratory capacity from the forced vital capacity at preheating. End‐inspiratory lung volume was calculated by adding *V*_T_ to the end‐expiratory lung volume. The work of breathing, end‐expiratory, and end‐inspiratory lung volumes were calculated by averaging data over five breaths.

### Statistical analysis

*V*_E_, *P*_ETCO2_, MCAV, and the work of breathing during hyperthermia were selected as main variables in the present study. The minimum sample size was calculated on the basis of 80% power and a significance level of 0.05 using controlled‐breathing‐related differences and standard deviations from our pilot experiments. The minimum sample sizes were estimated to be 5, 5, 4, and 8 for *V*_E_, *P*_ETCO2_, MCAV, and the work of breathing, respectively. The sample size used in this study (*n* = 10) was thus adequate for our analysis. Two‐factor repeated‐measures ANOVA was used to analyze the time‐dependent data using trial (levels: Normal‐breathing and Controlled‐breathing) and heating duration (levels: pre, 5, 10, 15, 20, 25, 30 min and end of heating) as factors. Two‐factor repeated‐measures ANOVA was also used to analyze the core temperature‐dependent data using trial (levels: Normal‐breathing and Controlled‐breathing) and *T*_es_ level (levels: 36.6, 37.0, 37.4, 37.8, 38.2, and 38.6°C) as factors. When a main effect or interaction was detected, a post hoc Bonferroni multiple comparisons test was performed to identify pairwise differences. Paired *t*‐tests were used to compare the heating time and body weight between the two trials. Pearson's product moment correlation coefficients were used to determine associations between variables. Eta‐squared (*η* ^2^) was calculated as a measure of effect size and interpreted as 0.01 = a small effect, 0.06 = a medium effect, 0.14 = a large effect (Cohen 1988). Data are reported as means ± SD. Values of *P* < 0.05 were considered statistically significant. All statistical analyses were performed using the SPSS statistics package (version 19.0, SPSS Inc.).

## Results

### Heating duration and body weight

Heating duration did not differ between trials (normal‐ vs. controlled‐breathing, 40.5 ± 6.9 vs. 39.5 ± 6.4 min, *P *=* *0.68). Body weight losses over the course of the experiment were similar in the normal‐ and controlled‐breathing trials (1.6 ± 1.0 vs. 1.4 ± 0.5% body weight loss, *P* = 0.29).

### Time‐dependent changes

#### Body temperatures, circulatory and respiratory responses

**Figure 1:**
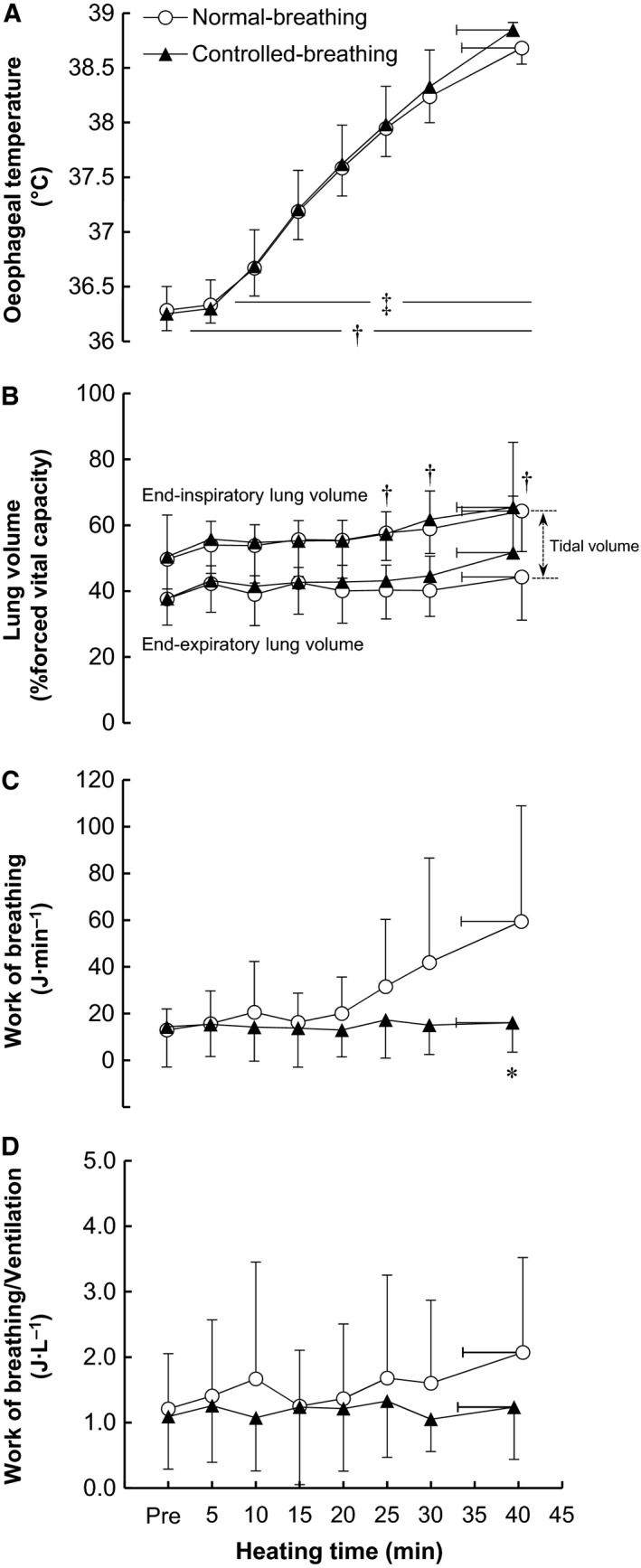
Time‐dependent changes in esophageal temperature (A), lung volume (B), work of breathing (C), and work of breathing/ventilation (D) during passive heating in the normal‐breathing and controlled‐breathing trials. Values are means ± SD. *P = 0.01, normal‐ versus controlled‐breathing; ^†^ *P *< 0.03, versus prior to heating (pre) in the normal‐breathing trial; ^‡^ *P *< 0.002, versus pre in the controlled‐breathing trial.

Time‐dependent changes in body temperature, circulatory and respiratory variables are shown in Table 1 and Figure 1A. *T*_es_ increased similarly during passive heating in both trials (*P* = 0.61, *η* ^2^ = 0.001 for a main effect of trial, Fig. 1A). The observed changes in mean skin temperature and heart rate were also similar in the two trials (*P* = 0.91 and 0.94, both *η* ^2^ = 0.001 for a main effect of trial, respectively). MAP was higher in the controlled‐breathing than the normal‐breathing trial after 30 min of heating (*P* < 0.005). MCAV was also higher in the controlled‐breathing trial after 25 min (*P* < 0.04), and CVC was higher in the controlled‐breathing trial at the end of heating (*P* < 0.001). A significant main effect of trial was detected for *V*_E_, *V*_T_, *f*,* P*_ETCO2_, *P*_ETO2_, *V*CO_2_ and the respiratory exchange ratio (*P* = 0.001, 0.01, 0.02, 0.001, <0.001, 0.004, and 0.03; *η* ^2^ = 0.10, 0.08, 0.06, 0.21, 0.23, 0.04, and 0.10, respectively) but not *V*O_2_ (*P* = 0.94, *η* ^2^ = 0.001). *V*_E_ was lower in the controlled‐breathing than the normal‐breathing trial during the latter half of heating due to lower *V*_T_ and *f* (all *P* < 0.04). *P*_ETCO2_ was higher in the controlled‐breathing trial after 20 min of heating (all *P* < 0.02). Difficulty of breathing increased during the heating with no difference between trials (*P* = 0.17, *η* ^2^ = 0.02 for a main effect of trial).

**Table 1:** Time‐dependent changes in circulatory and respiratory variables during normal‐breathing and controlled‐breathing trials

	Pre	5 min	10 min	15 min	20 min	25 min	30 min	End of heating
Heart rate, beats min^−1^								
Normal‐breathing	66 ± 7	69 ± 6	83 ± 9†	93 ± 10†	104 ± 12†	111 ± 12†	118 ± 13†	130 ± 15†
Controlled‐breathing	65 ± 5	70 ± 6‡	85 ± 8‡	95 ± 11‡	102 ± 10‡	110 ± 9‡	117 ± 9‡	128 ± 9‡
Mean arterial pressure, mmHg								
Normal‐breathing	91 ± 6	91 ± 7	92 ± 8	91± 7	92 ± 6	90 ± 6	90 ± 5	89 ± 7
Controlled‐breathing	87 ± 7	88 ± 6	89 ± 6	90 ± 5	92 ± 6	94 ± 8	96 ± 6* ^,^ ‡	98 ± 9* ^,^ ‡
Middle cerebral artery blood flow velocity, cm sec^−1^								
Normal‐breathing	65.0 ± 5.2	65.7 ± 5.9	62.0 ± 4.9	58.4 ± 5.5†	57.0 ± 5.1†	54.2 ± 6.3†	51.7 ± 8.4†	43.5 ± 5.1†
Controlled‐breathing	65.4 ± 6.8	64.4 ± 5.9	61.4 ± 6.2‡	59.4 ± 8.7	59.6 ± 6.4‡	59.7 ± 6.5*	60.0 ± 6.0*	60.5 ± 7.3*
Cerebral vascular conductance, cm sec^−1^ mmHg								
Normal‐breathing	0.72 ± 0.06	0.72 ± 0.08	0.69 ± 0.10	0.65 ± 0.09†	0.63 ± 0.08†	0.60 ± 0.09†	0.58 ± O.ll†	0.49 ± 0.08†
Controlled‐breathing	0.75 ± 0.08	0.74 ± 0.07‡	0.69 ± 0.08‡	0.66 ± 0.10‡	0.65 ± 0.08‡	0.64 ± 0.09‡	0.64 ± 0.08‡	0.63 ± 0.08* ^,^ ‡
Minute ventilation, L min^−1^								
Normal‐breathing	9.3 ± 0.8	10.1 ± 0.8	10.5 ± 1.3	10.9 ± 1.4	12.0 ± 1.3†	14.6 ± 3.5†	18.3 ± 6.7†	26.7 ± 6.2†
Controlled‐breathing	9.6 ± 0.5	10.4 ± 0.9	11.0 ± 1.3	11.4 ± 2.0	11.0 ± 1.1* ^,^ ‡	11.3 ± 1.7*	11.3 ± 1.0* ^,^ ‡	12.3 ± 1.7* ^,^ ‡
Tidal volume, mL								
Normal‐breathing	554 ± 57	553 ± 60	572 ± 79	587 ± 101	655 ± 137	755 ± 159†	760 ± 166	920 ± 203†
Controlled‐breathing	545 ± 76	567 ± 92	582 ± 97	614 ± 126	597 ± 79	612 ± 84*	626 ± 90*	652 ± 75* ^,^ ‡
Respiratory frequency, breaths min^–1^								
Normal‐breathing	17 ± 2	18 ± 2	19 ± 4	19 ± 5	19 ± 5	20 ± 7	24 ± 8	30 ± 8†
Controlled‐breathing	18 ± 3	19 ± 3	20 ± 4	19 ± 4	19 ± 4	19 ± 4	19 ± 4*	19 ± 4*
End‐tidal carbon dioxide pressure, mmHg								
Normal‐breathing	37.7 ± 2.2	38.0 ± 2.2	37.8 ± 2.4	36.6 ± 2.7	36.8 ± 2.1	34.0 ± 3.3	31.2 ± 5.5	22.7 ± 4.2†
Controlled‐breathing	38.5 ± 2.3	37.6 ± 2.3	37.8 ± 2.3	38.0 ± 3.0	39.0 ± 2.6*	39.3 ± 3.1*	39.8 ± 2.5*	39.3 ± 3.2*
Oxygen uptake, mL min^−1^								
Normal‐breathing	245 ± 33	257 ± 35	264 ± 42	278 ± 40†	306 ± 43†	326 ± 49†	341 ± 43†	358 ± 50†
Controlled‐breathing	250 ± 27	255 ± 32	269 ± 40	283 ± 43‡	303 ± 50‡	319 ± 55‡	337 ± 62‡	364 ± 56‡
Difficulty of breathing (*n* = 9)								
Normal‐breathing	1.3 ± 0.8	1.7 ± 1.1	2.3 ± 1.2	3.1 ± 0.8†	3.9 ± 1.1†	4.9 ± 1.2†	5.9 ± 1.8†	6.8 ± 1.6†
Controlled‐breathing	1.2 ± 0.8	1.7 ± 0.8	2.6 ± 0.9	3.7 ± 0.9‡	4.7 ± 1.1‡	6.1 ± 1.7‡	6.7 ± 2.0‡	8.4 ± 2.5‡

Values are means ± SD. *n* = 10 except for breathing effort.

* *P *<* *0.05 versus normal‐breathing trial.

† *P *<* *0.04 versus prior to heating (pre) in the normal‐breathing trial.

‡ *P *<* *0.05 versus pre in the controlled‐breathing trial.

#### Respiratory mechanics

**Figure 2:**
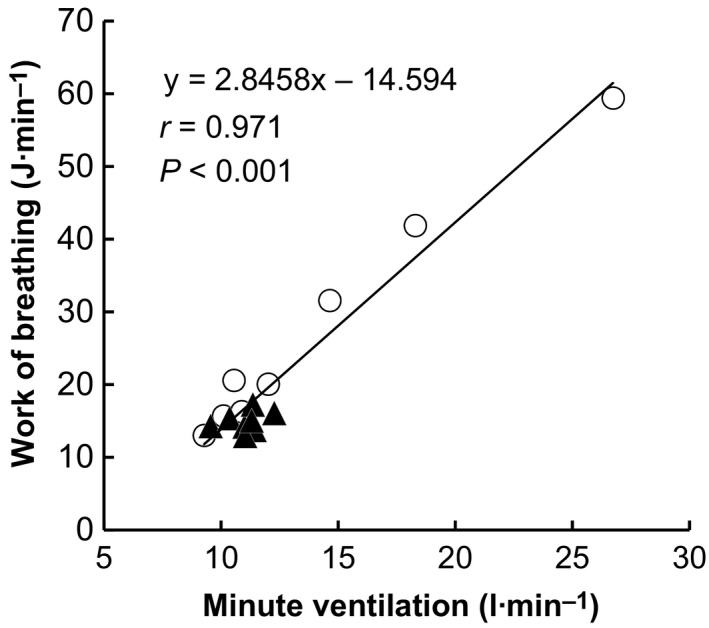
Relationship between minute ventilation and the work of breathing during passive heating in the normal‐breathing and controlled‐breathing trials. Values are expressed as the mean obtained every 5 min during heating.

Time‐dependent changes in respiratory mechanics are shown in Figure 1. Changes in end‐expiratory and end‐inspiratory lung volumes are presented in Figure 1B as percentages of forced vital capacity determined pre. End‐expiratory and inspiratory lung volumes did not differ between trials (*P* = 0.25 and 0.71, *η* ^2^ = 0.03 and 0.003 for a main effect of trial, respectively). End‐expiratory lung volume increased slightly during heating, but did not significantly change as compared to preheating. In the normal‐breathing trial, end‐inspiratory lung volume had increased significantly after 25 min relative to preheating (all *P* < 0.02). The work of breathing increased from 13.0 ± 8.9 at pre to 59.4 ± 49.5 J min^−1^ at the end of heating in the normal‐breathing trial, whereas in the controlled‐breathing trial it remained constant such that 16.1 ± 12.6 J min^−1^ was recorded at the end of heating (*P* = 0.01, Fig. 1C). The work of breathing per *V*_E_ was maintained nearly constant throughout the heating in either trial with no between‐trial difference (*P* = 0.49 and 0.13, *η* ^2^ = 0.04 and 0.03 for main effects of trial and heating duration, respectively) (Fig. 1D). In addition, the work of breathing pooled across the normal‐ and controlled‐breathing conditions correlated positively with *V*_E_ (*r* = 0.971, *P* < 0.001) (Fig. 2).

#### *T*_es_‐dependent changes in ventilatory and cerebrovascular responses

**Figure 3:**
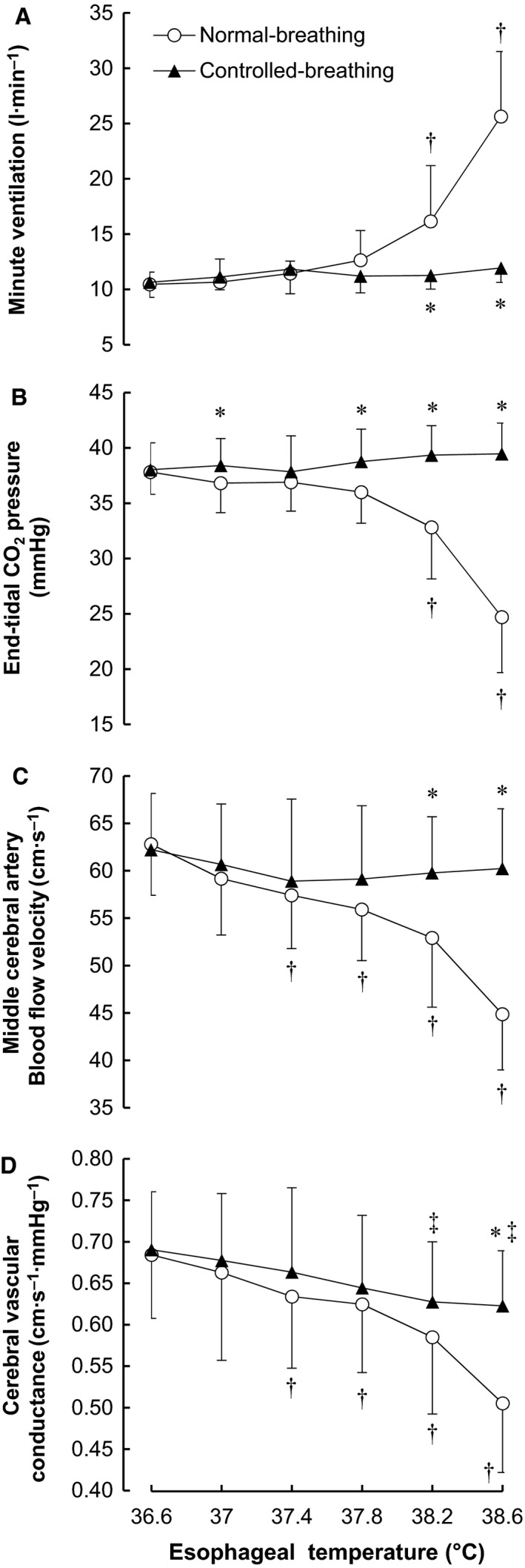
Esophageal temperature‐dependent changes in minute ventilation (A), end‐tidal CO_2_ pressure (B), middle cerebral artery blood flow velocity (C), and cerebral vascular conductance (D) during passive heating in the normal‐breathing and controlled‐breathing trials. Values are means ± SD. *P < 0.03, normal‐ versus controlled‐breathing; †*P *<* *0.04, versus 36.6°C in the normal‐breathing trial; ^‡^ *P* < 0.03, versus 36.6°C in the controlled‐breathing trial.

**Figure 4:**
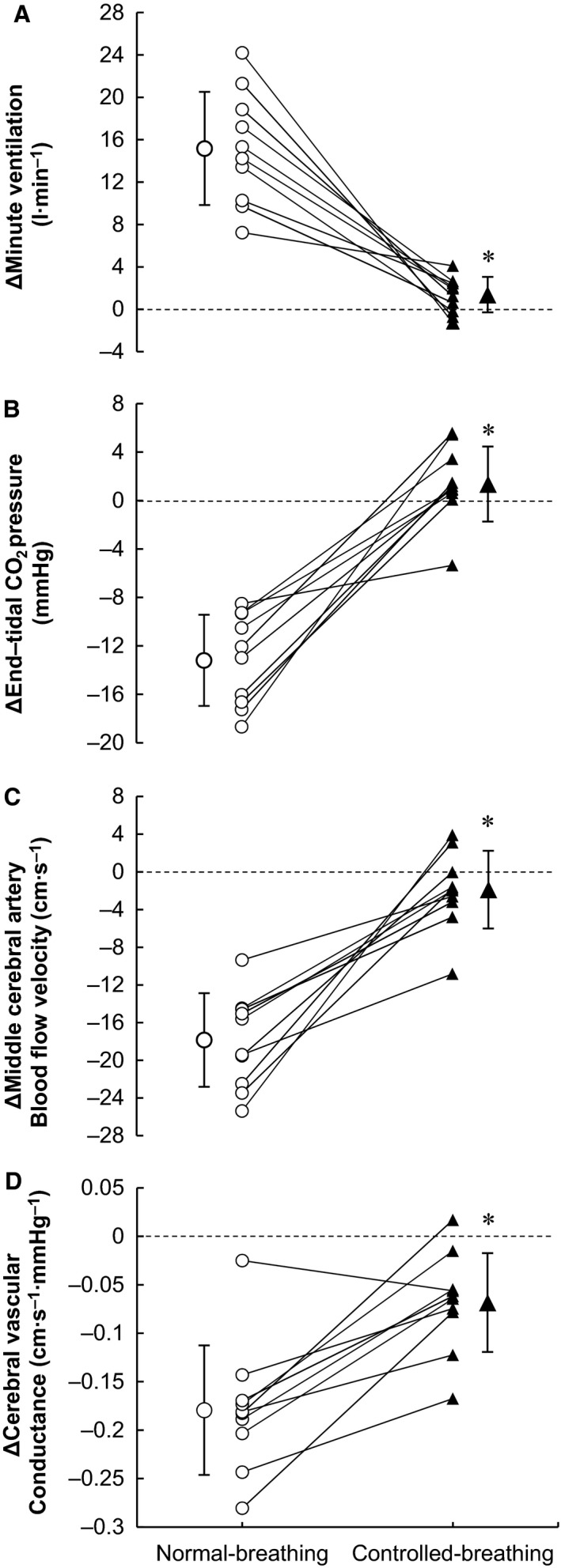
Changes (Δ) in minute ventilation (A), end‐tidal CO_2_ pressure (B), middle cerebral artery blood flow velocity (C), and cerebral vascular conductance (D) occurring as Esophageal temperature was raised from 36.6°C to 38.6°C. Mean and individual data are presented in both the normal‐breathing and controlled‐breathing trials. Values are means ± SD. *P < 0.001, normal‐breathing versus controlled‐breathing.

We compared the ventilatory and cerebrovascular responses elicited by the similar rise in *T*_es_ seen under the two conditions (Fig. 3). *V*_E_ was lower in the controlled‐breathing than normal‐breathing trial at *T*_es_ = 38.2–38.6°C (both *P* < 0.02). *P*_ETCO2_ was higher in the controlled‐breathing trial at *T*_es_ = 37.0°C and 37.8–38.6°C (all *P* < 0.01). MCAV and CVC were higher in the controlled‐breathing trial at *T*_es_ = 38.2–38.6°C (both *P* < 0.03) and 38.6°C (*P* = 0.02), respectively. Figure 4 illustrates the mean and individual data for the changes in *V*_E_, *P*_ETCO2_, MCAV, and CVC from *T*_es_ = 36.6°C to 38.6°C. Voluntary control of breathing diminished the increase in *V*_E_ and decreases in *P*_ETCO2_, MCAV, and CVC observed in the normal‐breathing trial at *T*_es_ = 38.6°C by 86 ± 19, 111 ± 36, 82 ± 31 and 58 ± 44%, respectively.

## Discussion

This study examined whether heat‐induced hyperventilation can be diminished through voluntary control of breathing, and, if so, how this affects respiratory mechanics, PaCO_2_, and MCAV during passive heating. We demonstrated for the first time that heat‐induced hyperventilation is accompanied by increases in the work of breathing chiefly due to increases in *V*_E_ without a change in end‐expiratory lung volume. This spontaneous hyperventilation elicited by passive heating can be nearly prevented by voluntary control of breathing, which attenuated largely the decreases in *P*_ETCO2_ and MCAV and the increases in the work of breathing that occurred during normal‐breathing.

### Ventilatory and cerebrovascular responses during hyperthermia at rest

Hyperthermia‐induced hyperventilation is perhaps caused by elevations in the temperatures of the brain (Chai and Lin 1972; Boden et al. 2000) and carotid chemoreceptors (Chu et al. 2007; Fujii et al. 2008a). Consistent with previous studies (Cabanac and White 1995; Fujii et al. 2008b; Tsuji et al. 2012a), we found that there was a *T*_es_ threshold for hyperventilation at 38.0 ± 0.3°C and that *V*_E_ rose along with increasing *T*_es_ at a rate of 30.4 ± 19.6 L∙min^−1^∙C^−1^ in the normal‐breathing trial. The increase in *V*_E_ reached significance at >38.2°C *T*_es_ (Fig. 3A), which is explained by increases in both *V*_T_ and *f* (Table 1) and is consistent with earlier findings (Gaudio and Abramson 1968; Baker et al. 1996; Fujii et al. 2008b; Tsuji et al. 2012a). The heat‐induced hyperventilation is involuntary, but we demonstrated that the large portion of this response could be voluntarily suppressed. That is, the time‐ and *T*_es_‐ dependent increases in *V*_E_, *V*_T_, and *f* during normal‐breathing were all suppressed (Figs. 3A and Table 1). Similarly, we recently reported that during prolonged exercise in the heat, most healthy men were able to suppress heat‐induced hyperventilation by voluntarily controlling breathing (Tsuji et al. 2015). Healthy men thus appear able to voluntarily suppress heat‐induced hyperventilation regardless of resting or exercising.

Consistent with earlier studies (Fan et al. 2008; Fujii et al. 2008a; Low et al. 2008; Brothers et al. 2009; Nelson et al. 2011; Bain et al. 2013; Ogoh et al. 2014), in the normal‐breathing trial MCAV and CVC decreased with rising *T*_es_ as compared to normothermia (Figs. 3C and D). These reductions in MCAV and CVC were largely suppressed in the controlled‐breathing trial, indicating that voluntary suppression of heat‐induced hyperventilation negates the heat‐induced reduction in cerebral blood flow. In the present study, voluntary breathing control reduced increases in *V*_E_ occurring at *T*_es_ = 38.6°C by 86% and the decreases in *P*_ETCO2_, MCAV, and CVC by 111%, 82%, and 58%, respectively. Given that the pattern of *P*_ETCO2_ response was similar to those of the cerebral vascular variables (i.e., MCAV and CVC), it seems likely that the higher MCAV and CVC during voluntary breathing control were partially a consequence of altering PaCO_2_. It should be noted that it is still debatable whether the reductions in MCAV during passive heating could be partially (Fujii et al. 2008a; Brothers et al. 2009) or entirely (Nelson et al. 2011; Bain et al. 2013) accounted for by the hyperventilation‐induced decreases in PaCO_2_. For instance, we recently reported that prevention of hypocapnia by CO_2_ gas inhalation throughout passive heating resulted in a partial restoration of MCAV (~36% of total reduction) (Tsuji et al. 2018). Regarding the hypocapnia‐independent factor(s), if at all, one of potential candidate is MAP. MAP in the normal‐breathing trial was maintained at the baseline level throughout the passive heating, though MAP in the controlled‐breathing trial gradually increased with voluntary suppression of hyperventilation. Consequently, MAP was ~10 mmHg higher at the end of heating than at pre or during the normal‐breathing trial, and CVC in the controlled‐breathing trial decreased gradually compared to preheating (Table 1). This suggests that increases in cerebral perfusion pressure associated with elevated MAP may have contributed to the higher MCAV during controlled‐breathing. The precise mechanism(s) underpinning the increases in MAP associated with voluntary suppression of breathing remain(s) elucidated.

Hyperventilation and hypoventilation both increase the sensation of dyspnea independently of PaCO_2_, with a greater effect during hypoventilation (Chonan et al. 1990). We therefore assumed that heat‐induced increases in *V*_E_ would themselves lead to increases in dyspnea in the normal‐breathing trial, and that the suppression of *V*_E_ in the controlled‐breathing would result in even greater increases in dyspnea. We found, however, that difficulty of breathing, an index of dyspneic sensation, similarly increased during passive heating in both trials (Table 1). Thus, voluntary suppression of *V*_E_ apparently does not cause further increases in feelings of dyspnea. It should be noted, however, that at the end of heating, difficulty of breathing tended to be higher in the controlled‐breathing than in the normal‐breathing trial (*P *=* *0.15), with seven of nine participants showing a higher score during controlled‐breathing. Taking these results into consideration, it seems reasonable to conclude that most healthy men can voluntarily suppress heat‐induced hyperventilation despite a greater sensation of discomfort.

Although nine of the 10 participants were able to largely (77–104%) suppress the heat‐induced hyperventilation, one individual was able to only partially (35%) suppress the response (Fig. 4). As a result, his *P*_ETCO2_, MCAV, and CVC were also only partially restored (by 20, 23, and 20%, respectively). In this individual, by the end of heating, difficulty of breathing had increased to level 4 (somewhat hard) in the normal‐breathing trial and to the maximum 10 (very, very hard) in the controlled‐breathing trial. This suggests individuals who have strong sensation of dyspnea may not be able to greatly suppress heat‐induced hyperventilation by voluntary control of breathing.

### Respiratory mechanics during hyperthermia at rest

This is, to our knowledge, the first study to report on the respiratory mechanics during hyperthermia. The work of breathing gradually increased and reached ~60 J min^−1^ as *V*_E_ reached ~27 L min^−1^ by the end of heating (Fig. 1C). By contrast, the work of breathing was maintained nearly constant throughout heating in the controlled‐breathing trial, indicating that voluntary suppression of heat‐induced hyperventilation markedly reduced the work of breathing. We also observed a significant correlative association between the work of breathing and *V*_E_ (Fig. 2), which is consistent with previous findings (Milici‐Emili and Petit 1960; Aaron et al. 1992a,b). The work of breathing–*V*_E_ relation is also reportedly curvilinear when evaluated at the wide range of *V*_E_ of ~30 to 150 L min^−1^ (Milici‐Emili and Petit 1960; Aaron et al. 1992a), but likely not at the low levels of *V*_E_ as observed in the present study. In addition, the work of breathing per ventilation remained unchanged throughout heating regardless of trials, and it was similar in both trials (Fig. 1D). We therefore conclude that the increased work of breathing associated with heat‐induced hyperventilation is mainly caused by the increase in ventilation per se.

We also showed that end‐expiratory lung volume was maintained at normothermic levels throughout the passive heating in both trials (Fig. 1B). This suggests that end‐expiratory lung volume is unaffected by elevations in the core temperature of ~2.5°C and the resultant heat‐induced hyperventilation, and that voluntary suppression of the hyperventilation does not affect the lung volume. Butler and Arnott (1955) previously reported that the work of breathing increases with decreases or increases in end‐expiratory lung volume from the normal level, though such an effect of lung volume level on the work of breathing may not have occurred in the present study. Furthermore, it is well known that exercise decreases end‐expiratory lung volume below the resting level (Henke et al. 1988; Guenette et al. 2007), though the present result infers that there is little linkage between this reduction in end‐expiratory lung volume and the elevation in core temperature observed during exercise.

We previously reported slight but significant decreases in *V*O_2_ with voluntary suppression of the gradual increase in *V*_E_ during prolonged exercise in the heat (Tsuji et al. 2015). This suggests that hyperthermia‐induced hyperventilation increases the work of breathing, and thus the oxygen cost (though we did not measure that in the work). Unlike in our earlier report (Tsuji et al. 2015), we found in the present study that *V*O_2_ did not differ between the two trials (Table 1), suggesting that *V*O_2_ is unaffected by hyperthermia‐induced hyperventilation during passive heating. Assuming a 1‐L increase in *V*_E_ leads to an approximately 1.2 mL increase in *V*O_2_ under resting conditions (Lorenzo and Babb 2012), the difference in *V*_E_ between the normal‐ and controlled‐breathing trials at the end of heating (~13 L∙min^−1^) would increase *V*O_2_ by about 16 mL, which appears to be too small to accurately detect. Another possible explanation for the disparate findings is the different levels of *V*_E_. A prior study reported that the work of breathing increases exponentially with increases in *V*_E_, whereas respiratory muscle *V*O_2_ increases linearly with increases in the work of breathing (Aaron et al. 1992a). This implies that the effects of suppressing *V*_E_ on the work of breathing and respiratory muscle VO_2_ are more pronounced during exercise, which is accompanied by higher *V*_E_ levels, than during passive heating, where *V*_E_ is low. Furthermore, it is worth mentioning that increases in the work of breathing can activate the muscle metaboreflex in the respiratory muscles, leading to locomotor muscle vasoconstriction. However, an earlier study reported that this response is only observed when exercise intensity is greater than 75% *V*O_2max_, during which the levels of ventilation, and thus the work of breathing, are substantially elevated (Dempsey et al. 2006). Because breathing levels are much lower during hyperthermia at rest than during intense exercise, it seems unlikely that a robust blood distribution associated with increased work of breathing occurred during the passive hyperthermia at rest in the present study. However, future study is warranted to directly test this possibility.

### Limitation

The present study and our earlier one (Tsuji et al. 2015) used a protocol in which participants controlled both *f* and *V*_T_ as much as possible with the aid of feedback. However, it remains uncertain whether individuals can voluntarily suppress hyperthermia‐induced hyperventilation without feedback for either *f* or *V*_T_, or both. We also do not know whether suppression of hyperthermia‐induced hyperventilation, with and without feedback, differentially modulates PaCO_2_, cerebral blood flow, and the work of breathing. We also used MCAV as an index of cerebral blood flow, though a recent magnetic resonance imaging study reported that MCA diameter decreased by 4% when *P*_ETCO2_ decreased from 36 to 23 mmHg (Coverdale et al. 2014). This is in contrast to earlier reports that the diameter was unchanged by decreases in *P*_ETCO2_ to ~25 mmHg (Giller et al. 1993; Serrador et al. 2000). It is therefore possible that we underestimated the magnitude of the MCAV reduction in the normal‐breathing trial, though MCAV in the controlled‐breathing trial reflected blood flow without an effect of diameter. In addition, it remains unclear whether hyperthermia itself modulates MCA diameter independently of hypocapnia.

### Perspectives and significance

Decreases in PaCO_2_ and cerebral blood flow during hyperthermia reportedly lead to increases in brain temperature (Nybo et al. 2002) and central fatigue (Ross et al. 2012). Ross et al. (2012) reported that the decrease in cortical activation seen during passive heating is related to heat‐induced hypocapnia and subsequent cerebral hypoperfusion. Our results suggest these detrimental effects can be mitigated somewhat through voluntary control of breathing that restores PaCO_2_. Additional research will be required to test this possibility, however. Nonetheless, voluntary control of breathing could be an effective countermeasure to suppress hypocapnia and cerebral hypoperfusion during hyperthermia, along with the previously reported whole‐body skin cooling (Wilson et al. 2002; Lucas et al. 2010).

## Conclusion

In summary, heat‐induced hyperventilation during passive heating is accompanied by increases in the work of breathing reflecting increases in ventilation, per se, and occurs without changing end‐expiratory lung volume. This hyperventilatory response can be greatly suppressed in healthy young men through voluntary control of breathing with ventilatory feedback, mitigating the increased work of breathing and decreased arterial CO_2_ pressure and cerebral blood flow velocity observed during normal‐breathing.

## Conflict of Interest

None declared.
